# Supplementary material for: Genome-Wide and 16S rRNA Sequencing-Based Analysis on the Health Effects of Lacticaseibacillus paracasei XLK401 on Chicks
Source: Microorganisms. 2023 Aug 23;11(9):2140. doi: 10.3390/microorganisms11092140 (PMC10538037; doi:10.3390/microorganisms11092140)
Supplement: Supplementary file 1 [file microorganisms-11-02140-s001.zip › Supplementary Figure S1.pdf]

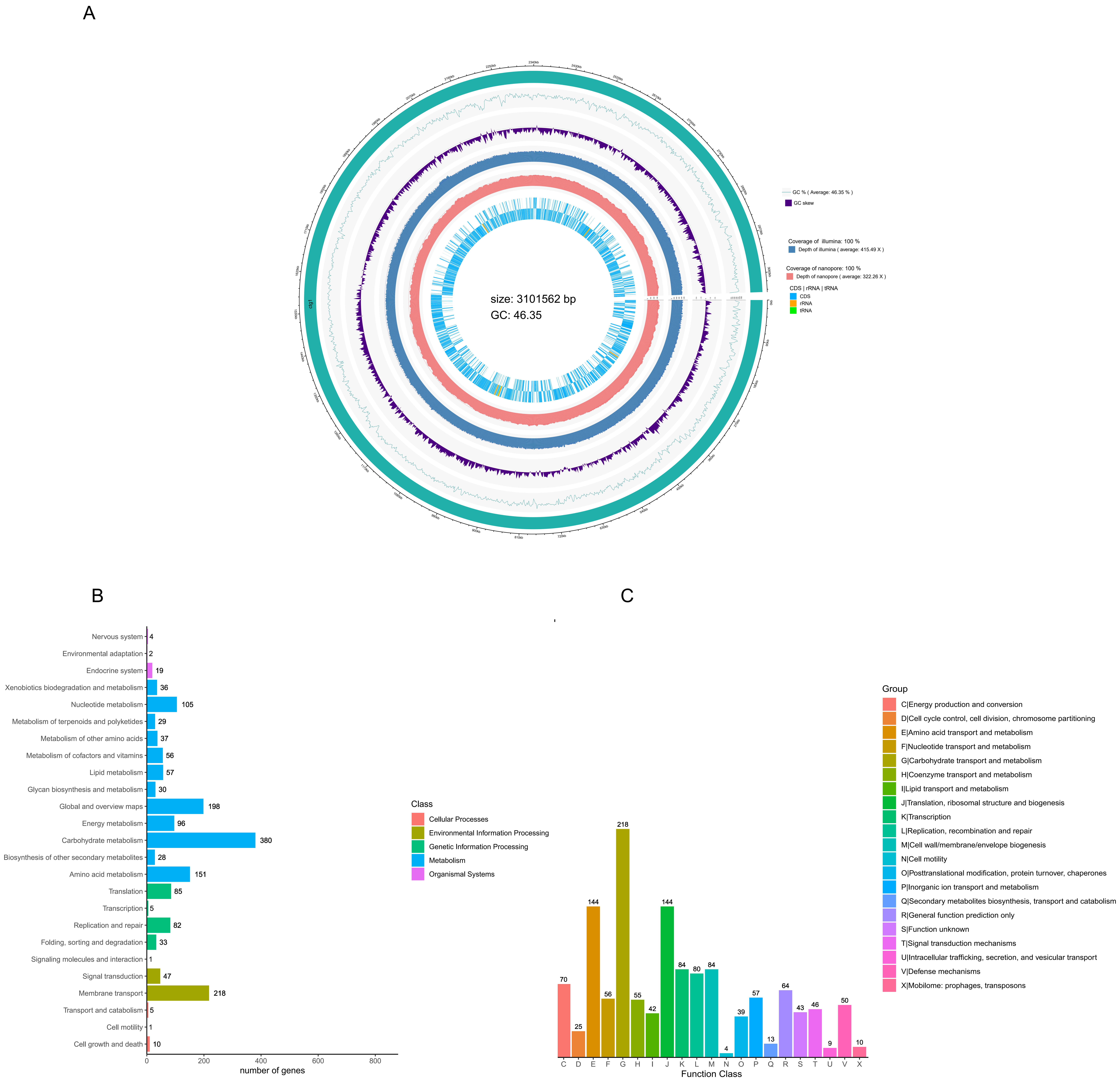
A
size: 3101562 bp
GC: 46.35
GC % (Average: 46.35 %)
GC skew
Coverage of illumina: 100 %
Depth of illumina (average: 415.49 X)
Coverage of nanopore: 100 %
Depth of nanopore (average: 322.26 X)
CDS | rRNA | tRNA
CDS
rRNA
tRNA
B
Nervous system 4
Environmental adaptation 2
Endocrine system 19
Xenobiotics biodegradation and metabolism 36
Nucleotide metabolism 105
Metabolism of terpenoids and polyketides 29
Metabolism of other amino acids 37
Metabolism of cofactors and vitamins 56
Lipid metabolism 57
Glycan biosynthesis and metabolism 30
Global and overview maps 198
Energy metabolism 96
Carbohydrate metabolism 380
Biosynthesis of other secondary metabolites 28
Amino acid metabolism 151
Translation 85
Transcription 5
Replication and repair 82
Folding, sorting and degradation 33
Signaling molecules and interaction 1
Signal transduction 47
Membrane transport 218
Transport and catabolism 5
Cell motility 1
Cell growth and death 10
0
200
400
600
800
number of genes
Class
Cellular Processes
Environmental Information Processing
Genetic Information Processing
Metabolism
Organismal Systems
C
70 25 144 56 218 55 42 144 84 80 84 4 39 57 13 64 43 46 9 50 10
C D E F G H I J K L M N O P Q R S T U V X
Function Class
Group
C|Energy production and conversion
D|Cell cycle control, cell division, chromosome partitioning
E|Amino acid transport and metabolism
F|Nucleotide transport and metabolism
G|Carbohydrate transport and metabolism
H|Coenzyme transport and metabolism
I|Lipid transport and metabolism
J|Translation, ribosomal structure and biogenesis
K|Transcription
L|Replication, recombination and repair
M|Cell wall/membrane/envelope biogenesis
N|Cell motility
O|Posttranslational modification, protein turnover, chaperones
P|Inorganic ion transport and metabolism
Q|Secondary metabolites biosynthesis, transport and catabolism
R|General function prediction only
S|Function unknown
T|Signal transduction mechanisms
U|Intracellular trafficking, secretion, and vesicular transport
V|Defense mechanisms
X|Mobilome: prophages, transposons
