# Supplementary figures and images for: Genome-Wide and 16S rRNA Sequencing-Based Analysis on the Health Effects of Lacticaseibacillus paracasei XLK401 on Chicks
Source: Microorganisms. 2023 Aug 23;11(9):2140. doi: 10.3390/microorganisms11092140 (PMC10538037; doi:10.3390/microorganisms11092140)

A

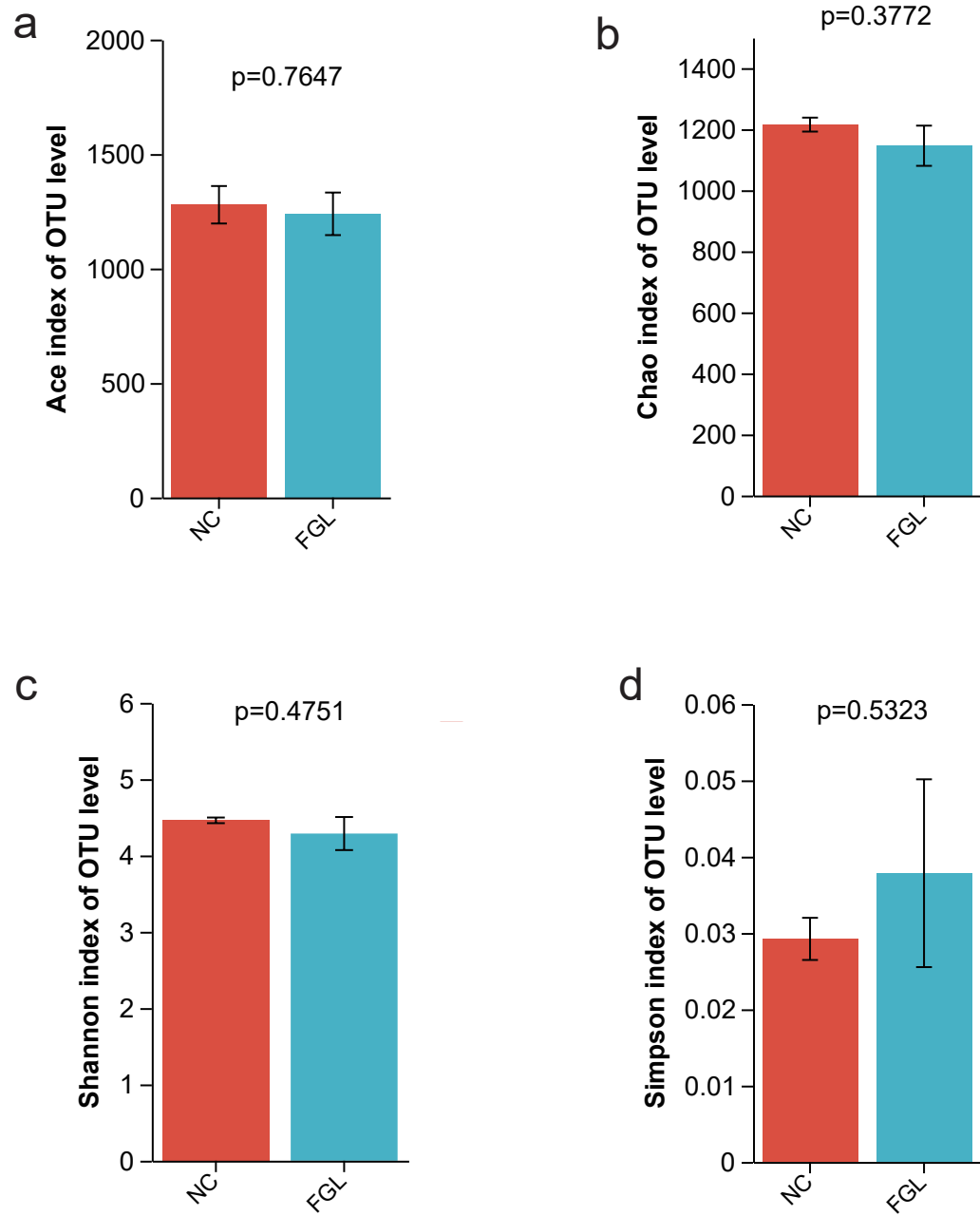

B

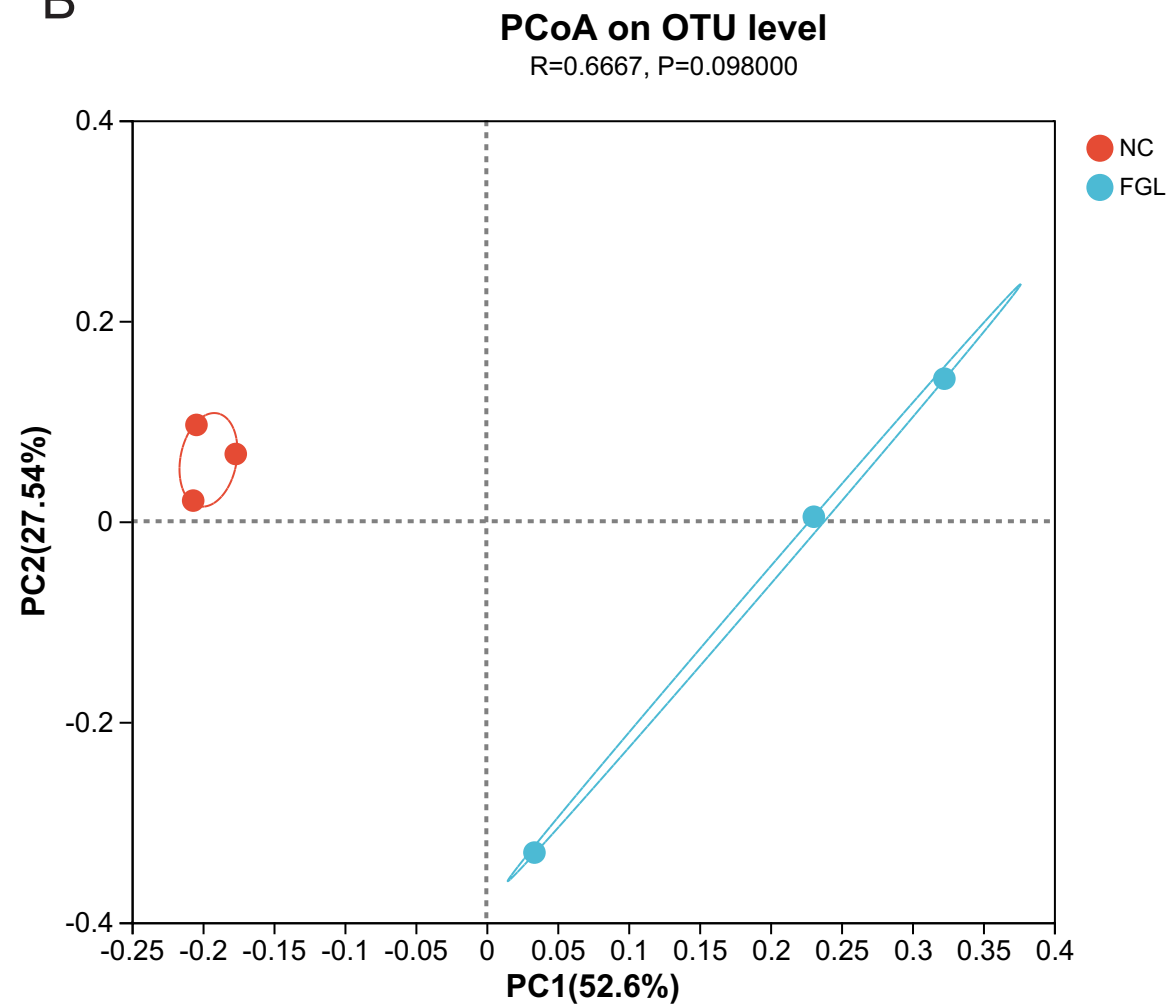

Supplement: Supplementary file 1 [file microorganisms-11-02140-s001.zip › Supplementary Figure S2.pdf]
